# Triggering the Amphotericin B Pore-Forming Activity by Phytochemicals

**DOI:** 10.3390/membranes13070670

**Published:** 2023-07-14

**Authors:** Svetlana S. Efimova, Anna I. Malykhina, Olga S. Ostroumova

**Affiliations:** Laboratory of Membrane and Ion Channel Modeling, Institute of Cytology of Russian Academy of Sciences, Tikhoretsky 4, 194064 Saint Petersburg, Russia

**Keywords:** lipid bilayers, ion-permeable pores, antibiotic, amphotericin B, polyphenols, alkaloids

## Abstract

The macrolide polyene antibiotic amphotericin B (AmB), remains a valuable drug to treat systemic mycoses due to its wide antifungal activity and low probability of developing resistance. The high toxicity of AmB, expressed in nephropathy and hemolysis, could be partially resolved by lowering therapeutic AmB concentration while maintaining efficacy. This work discusses the possibility of using plant polyphenols and alkaloids to enhance the pore-forming and consequently antifungal activity of AmB. We demonstrated that phloretin, phlorizin, naringenin, taxifolin, quercetin, biochanin A, genistein, resveratrol, and quinine led to an increase in the integral AmB-induced transmembrane current in the bilayers composed of palmitoyloleoylphosphocholine and ergosterol, while catechin, colchicine, and dihydrocapsaicin did not practically change the AmB activity. Cardamonin, 4′-hydroxychalcone, licochalcone A, butein, curcumin, and piperine inhibited AmB-induced transmembrane current. Absorbance spectroscopy revealed no changes in AmB membrane concentration with phloretin addition. A possible explanation of the potentiation is related to the phytochemical-produced changes in the elastic membrane properties and the decrease in the energy of formation of the lipid mouth of AmB pores, which is partially confirmed by differential scanning microcalorimetry. The possibility of AmB interaction with cholesterol in the mammalian cell membranes instead of ergosterol in fungal membranes, determines its high toxicity. The replacement of ergosterol with cholesterol in the membrane lipid composition led to a complete loss or a significant decrease in the potentiating effects of tested phytochemicals, indicating low potential toxicity of these compounds and high therapeutic potential of their combinations with the antibiotic. The discovered combinations of AmB with plant molecules that enhance its pore-forming ability in ergosterol-enriched membranes, seem to be promising for further drug development in terms of the toxicity decrease and efficacy improvement.

## 1. Introduction

Fungal invasive infections are associated with a wide range of side effects and high mortality rates, especially in immunocompromised patients [1,2,3,4]. Amphotericin B (AmB) remains the gold standard in the therapy of serious systemic fungal diseases due to its broad activity spectrum, low resistance, and high clinical efficacy [5,6,7] in various cases including post-COVID fungal infections [8].

AmB belongs to the polyene macrolides with a fungicidal mechanism of action accomplished mainly through pore formation in the membranes of fungal cells by the assembly of antibiotic and ergosterol (ERG) molecules, which induces outflow of electrolytes and other substances from the intracellular medium [9,10,11,12]. In planar lipid bilayers, addition of AmB on one side leads to the formation of predominantly cation-selective pores [13,14]. Using solid-state nuclear magnetic resonance spectroscopy and molecular dynamics simulations, the three-dimensional structure of the AmB pore in the bilayers formed from palmitoyloleoylphosphocholine (POPC) and ERG has been resolved as a single-length heptamer assembly [15]. The possibility of AmB interaction with mammalian sterol—cholesterol (CHOL) determines the high toxicity of the antibiotic, which seriously limits clinical use of AmB [16].

Chemosensitization by natural compounds, to increase the effectiveness of commercial antimycotics is a promising approach to overcoming pathogen resistance, reducing side effects and the cost of antifungal treatment, especially when bearing in mind the high cost of bringing a new antimycotic drug to the pharmaceutical market [17]. Thus, one way to reduce AmB toxicity is to potentiate its antifungal action with other molecules, so that less active substance is needed to achieve the same effect. In this regard, the naturally occurring polyphenols and alkaloids are of particular interest. Alves et al. [18] demonstrated the potential effect of combining AmB with the phenolic compounds, gallic and ellagic acids, in pharmaceutical formulations for the treatment of cutaneous leishmaniasis in mice. Two other phenolic compounds, ethyl cumarate and (1E)-1-(4-hydroxyphenyl)hex-1-en-3-one, in combination with AmB showed a 16 times decrease in the fungicidal concentration of AmB, without noticeable cytotoxicity to mammalian cells [19]. Anthraquinones extracted from *Aloe* spp., emodin, barbaloin, and chrysophanol, have shown pharmacological synergism with AmB at sub-inhibitory concentrations against *Cryptococcus neoformans* [20]. Moreover, the authors suggested that the anthraquinones enhanced the pore formation by AmB. Nidhi et al. demonstrated that 2-β-pinene, δ-3 carene, and D-limonene from *Citrus aurantium* essential oil exhibited synergism with AmB against *Candida albicans* and increased its antifungal efficacy by more than 8–30 times [21]. According to [22], quercetin protects red blood cells from AmB-mediated toxicity and decreases the MIC for *C. neoformans* from 0.25 μg/mL for AmB alone, to 0.125 μg/mL for the combination. Combined treatment with catechin and AmB showed a marked decrease in the growth of AmB-resistant *C. albicans* and a two-fold decrease in MIC for the susceptible strain [23]. The synergistic effect of resveratrol and AmB was studied against *Leishmania amazonensis* [24]. The synergistic interaction of eugenol and AmB was demonstrated [25]. Two indole alkaloids, shearinines D and E, were shown to enhance the AmB potency by eight times against clinical *Candida* spp. including the prevention of the biofilm formation [26]. A series of synthetic analogs of a pyrrolo-quinoline alkaloid, camptothecin, demonstrate the synergistic antifungal activity with AmB against yeasts [27]. Despite the fact that the abovementioned small molecules successfully potentiated AmB activity, no mechanism of action was studied or even proposed for such findings. Therefore, the search for AmB potentiators seems to be random and inefficient. Understanding the underlying principle of similar action shown by chemically different molecules could pave the path for a targeted and highly productive approach for AmB improvement and antibiotic development.

**Figure 1:**
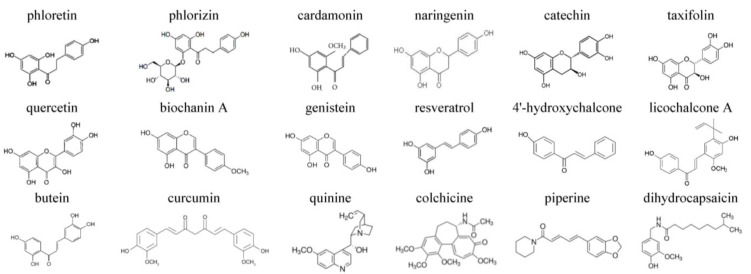
Chemical structures of tested phytochemicals (phloretin, phlorizin, cardamonin, naringenin, catechin, taxifolin, quercetin, biochanin A, genistein, resveratrol, 4′-hydroxychalcone, licochalcone A, butein, and curcumin) and alkaloids (quinine, colchicine, piperine, and dihydrocapsaicin).

In this work, we tested the idea that small molecules of plant origin can potentiate the pore-forming ability of AmB by modulating membrane lipid media. Plant polyphenols, phloretin, phlorizin, cardamonin, naringenin, catechin, taxifolin, quercetin, biochanin A, genistein, resveratrol, 4′-hydroxychalcone, licochalcone A, butein, and curcumin, and alkaloids, quinine, colchicine, piperine, and dihydrocapsaicin, were tested. Figure 1 demonstrates the chemical structure of the tested phytochemicals. To predict the possible selectivity index, lipid bilayers enriched with ERG and CHOL mimicking the fungal and mammalian cell membranes were used. The discovered combinations of AmB with phytochemicals seem to be promising for developing innovative lipid formulations of the antibiotic.

## 2. Materials and Methods

### 2.1. Materials

1-Palmitoyl-2-oleoyl-*sn*-glycero-3-phosphocholine (POPC) and cholesterol (CHOL) were purchased from Avanti Polar Lipids (Avanti Polar Lipids, Inc., Alabaster, AL, USA). Amphotericin B (AmB), ergosterol (ERG), KCl, HEPES, KOH, EDTA, dimethylsulfoxide (DMSO), methanol, chloroform, polyphenols (phloretin (≥99%, HPLC), phlorizin (≥99%, HPLC), cardamonin (≥98%, HPLC), naringenin (≥95%, HPLC), catechin (≥98%, HPLC), taxifolin (≥90%, HPLC), quercetin (≥95%, HPLC), biochanin A (≥95%, HPLC), genistein (≥98%, HPLC), resveratrol (≥99%, HPLC), 4′-hydroxychalcone (≥99%, HPLC), licochalcone A (≥96%, HPLC), butein (≥98%, HPLC), curcumin (≥80%, HPLC)), and alkaloids (quinine (≥98%, HPLC), colchicine (≥95%, HPLC), piperine (≥97%, HPLC), and dihydrocapsaicin (≥85%, HPLC)) were purchased from Sigma-Aldrich Company Ltd. (Gillingham, UK). Membrane bathing solutions of 0.15 M KCl (10 mM HEPES, pH 7.4) and 2.0 M KCl (10 mM HEPES, pH 7.4) were used. All experiments were performed at 25 ± 1 °C.

### 2.2. Electrophysiological Assay: The Formation of the Planar Lipid Bilayers and Their Modification by an Antibiotic and Phytochemicals

The planar lipid bilayers without solvent lenses were constructed according to the Montal and Mueller technique [28] on the aperture in the Teflon film which separates the Teflon chamber into two distinct compartments (*in* and *out*). The aperture was preliminarily treated with hexadecane. The planar lipid bilayers were composed of mixtures of 67 mol.% POPC and 33 mol.% sterol (ERG or CHOL) and bathed in 2.0 M KCl (pH 7.4). After the POPC/ERG (67/33 mol.%) and POPC/CHOL (67/33 mol.%) bilayers were completely stabilized in time, AmB was added from a 10 mM stock solution in DMSO to the *in*-compartment up to concentrations in the ranges of 0.5–8 and 3–10 µM, respectively, to obtain an integral current (more than 50 pA). The AmB concentrations were chosen according to [14].

**Figure 2:**
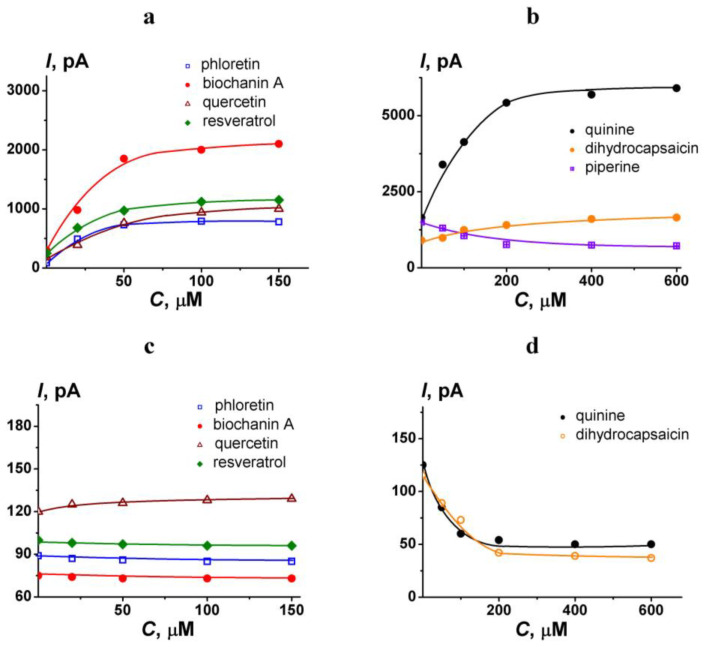
The dependence of transmembrane current induced by amphotericin B on the concentration of different polyphenols (**a**,**c**) and alkaloids (**b**,**d**). Membranes were formed from POPC/ERG (67/33 mol.%) (**a**,**b**) and POPC/CHOL (67/33 mol.%) (**c**,**d**) and bathed in 2.0 M KCl pH 7.4. The transmembrane voltage was 50 mV.

To modify the pore-forming ability of AmB, polyphenols and alkaloids were introduced into both chamber compartments up to 100 and 400 μM, respectively. The concentrations were chosen to maximize the effects of phytochemicals on the pore-forming antibiotic activity based on the obtained dependencies of AmB-induced transmembrane current on their concentration. Figure 2 presents the examples of *I*(*C*)-curves for different polyphenols (a) and alkaloids (b) in POPC/ERG (67/33 mol.%) and POPC/CHOL (67/33 mol.%) bilayers. An increase in polyphenol and alkaloid concentrations from 100 to 150 and from 400 to 600 μM, respectively, does not lead to an increase in their effects. Reduction in the concentrations of polyphenols and alkaloids up to 25 and 100 μM, respectively, is impractical due to the diminishing of the effects.

Tested phytochemicals alone do not alter the ion permeability of model lipid membranes at concentrations used within 120 min. Solvent (DMSO or ethanol) alone did not cause any changes in the stability and ion permeability of the model lipid membranes at the concentrations used (≤10^−4^ mg/mL).

The variation of the AmB pore-forming activity upon the introduction of phytochemicals was assessed as the ratio of the AmB-induced current flowing through the POPC/ERG (67/33 mol.%) or POPC/CHOL (67/33 mol.%) bilayer at a transmembrane voltage equal to 50 mV after (*I_m_*) and before the addition of plant molecule (*I_o_*). The *I_m_/I_o_*-values were averaged from three to seven independent experiments and are presented as mean ± S.E. (*p* ≤ 0.05).

The measurements of the transmembrane current (*I*) and the applied transmembrane voltage (*V*) were produced using Ag/AgCl electrodes with 2 M KCl/1.5% agarose bridges. At positive voltage the *in*-side compartment was positive with respect to the *out*-side.

The Axopatch 200B amplifier (Molecular Devices, LLC, Orleans Drive, Sunnyvale, CA, USA) in the voltage-clamp mode was used to measure the current. The signals were digitized by Digidata 1440A (Molecular Devices, LLC, Orleans Drive, Sunnyvale, CA, USA) at a 5 kHz sampling frequency using 1 kHz low-pass filtering. The data analysis was performed using pClamp 10.2 (Molecular Devices, LLC, Orleans Drive, Sunnyvale, CA, USA) and filtering by an 8-pole Bessel 100 kHz and Origin 8.0 (OriginLab Corporation, Northampton, MA, USA). 

### 2.3. Absorbance Spectroscopy Assay: The Formation of the Polyene-Loaded Lipid Vesicles and Their Modification by Phloretin

The technique of [29] with modifications for the formation of the polyene-loaded liposomes was used. Lipid mixture (67 mol.% POPC and 33 mol.% sterols (CHOL or ERG) without or with AmB and/or phloretin were suspended in a mixture of chloroform (67 vol.%) and methanol (33 vol.%). Equimolar contents of lipid, AmB and flavonoids (phloretin, biochanin A, and genistein) were used. The resulting solution was evaporated in a vacuum rotary evaporator at room temperature for 120 min. Further, the lipid film was dispersed in a buffer (0.15 M KCl, 10 mM HEPES, 1 mM EDTA, pH 7.4) and was exposed to ultrasound for 10 min. The AmB absorbance spectrum in the lipid microenvironment in the absence and presence of phytochemicals revealed distinct maxima at 363 nm and 427 nm that can be referred to self-associated and monomeric membrane forms of antibiotic [30]. The optical density values were averaged from two to four independent experiments and presented as mean ± S.E. (*p* ≤ 0.05).

Absorbance spectroscopy was determined using a spectrofluorometer “Fluorat-02-Panorama” (Lumex, Saint-Petersburg, Russia). All liposome suspensions were scanned from 300 to 450 nm with a 1 nm step. The extinction coefficient of AmB in DMSO was determined experimentally as 1.1 × 10^5^ M^−1^cm^−1^ and was in good agreement with the literature data [30,31].

### 2.4. Differential Scanning Microcalorimetry

Differential scanning microcalorimetry experiments were performed by a μDSC 7EVO microcalorimeter (Setaram, Caluire-et-Cuire, France). Giant unilamellar vesicles compose of DPPC were prepared by the electroformation method using Vesicle Pre Pro^@^ (Nanion Technologies, Munich, Germany) (standard protocol, 3 V, 10 Hz, 58 min, 55 °C). The resulting DPPC liposome suspension contained 2.5 mM lipid and was buffered by 5 mM HEPES at pH 7.4. The molar ratio of lipid:phlorizin and lipid:biochanin A was equal to 10:1. The liposomal suspension was heated and cooled at a constant rate of 0.2 and 0.3 °C/min, respectively. The reversibility of the thermal transitions was assessed by reheating the sample immediately after the cooling step from the previous scan. The temperature dependence of the excess heat capacity was analyzed using Calisto Processing (Setaram, Caluire-et-Cuire, France).

The peaks on the thermograms were characterized by the maximum temperature of the main phase transition (*T_m_*) of DPPC and the width of the main peak, i.e., the temperature difference between the upper (onset) and lower (completion) boundary of the main phase transition (Δ*T_b_*) of DPPC.

The values of Δ*T_m_* and ΔΔ*T_b_* were averaged from two independent experiments and presented as mean ± S.E. (*p* ≤ 0.05).

## 3. Results and Discussion

To mimic the action of AmB on fungal cell membranes the model lipid membranes were constructed from the binary mixture of POPC with the major fungal sterol, ERG [32,33]. The effects of polyphenols (phloretin, phlorizin, cardamonin, naringenin, catechin, taxifolin, quercetin, biochanin A, genistein, resveratrol, 4′-hydroxychalcone, licochalcone A, butein, and curcumin) and alkaloids (quinine, colchicine, piperine, and dihydrocapcaicin) on the AmB pore-forming activity in the membranes composed of POPC and ERG (67/33 mol.%) were tested.

**Figure 3:**
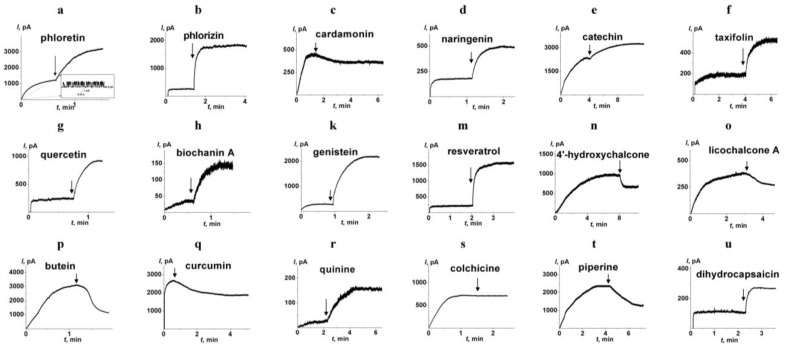
The effects of different phytochemicals on the pore-forming activity of amphotericin B. Polyphenols (**a**–**q**) and alkaloids (**r**–**u**) were added up to the concentration of 100 and 400 µM, respectively. The arrows indicate the moments of addition of phloretin (**a**), phlorizin (**b**), cardamonin (**c**), naringenin (**d**), catechin (**e**), taxifolin (**f**), quercetin (**g**), biochanin A (**h**), genistein (**k**), resveratrol (**m**), 4′-hydroxychalcone (**n**), licochalcone A (**o**), butein (**p**), curcumin (**q**), quinine (**r**), colchicine (**s**), piperine (**t**), and dihydrocapsaicin (**u**) to the membrane bath solution (2.0 M KCl, pH 7.4). The membranes were formed from POPC/ERG (67/33 mol.%). The antibiotic was added at the initial time moment and their concentration in bathing solution was equal to 5 (**a**), 2 (**b**), 3 (**c**), 1 (**d**), 7 (**e**), 3 (**f**), 2 (**g**), 1 (**h**), 1 (**k**), 2 (**m**), 5 (**n**), 5 (**o**), 8 (**p**), 5 (**q**), 1 (**r**), 3 (**s**), 7 (**t**), and 3 µM (**u**). The transmembrane voltage was 50 mV. Inset*:* The fluctuations of the transmembrane current caused by openings and closures of single ion-permeable pores produced by amphotericin B.

Figure 3 presents the kinetics of transmembrane current produced by AmB before and after the addition of the phytochemicals. The inset in Figure 3a demonstrates the fluctuations in single AmB pores at the initial time moment. Figure 3 shows that the addition of phloretin (3a), phlorizin (3b), naringenin (3d), taxifolin (3f), quercetin (3g), biochanin A (3h), genistein (3k), resveratrol (3m), and quinine (3r) led to significant improvement of pore-forming activity of AmB expressed during the increase in transmembrane current. The addition of catechin (3e) and dihydrocapsaicin (3u) slightly increased AmB pore-forming activity. Colchicine (3s) did not practically modulate AmB-produced current. Interestingly, the addition of the cardamonin (3c), 4′-hydroxychalcone (3n), licochalcone A (3o), butein (3p), curcumin (3q) and piperine (3t) caused a decrease in the AmB-produced transmembrane current. Table 1 presents the mean ratios between trans-bilayer currents induced by the antibiotic after and before the addition of tested phytochemicals (*I_m_/I_o_*). *I_m_/I_o_*-value increased in the following order: butein ≈ piperine ≈ curcumin ≈ cardamonin ≈ 4′-hydroxychalcone ≈ licochalcone A (*I_m_/I_o_* varies in the range from 0.4 to 0.7) < colchicine (*I_m_/I_o_* is about 1) < catechin ≈ dihydrocapsaicin (*I_m_/I_o_* is about 2) < naringenin ≈ taxifolin (*I_m_/I_o_* is about 3) ≤ phlorizin ≈ quinine (*I_m_/I_o_* is 4 ÷ 5) ≤ quercetin ≈ resveratrol (*I_m_/I_o_* is 6 ÷ 7) ≤ genistein ≈ biochanin A ≈ phloretin (*I_m_/I_o_* is about 10 ÷ 18). 

**Figure 4:**
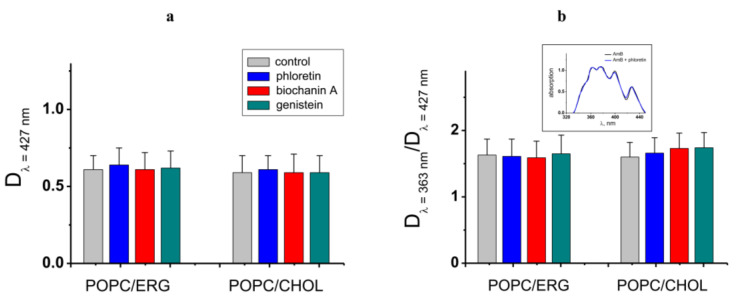
(**a**) Amphotericin B absorbance at 427 nm characterizing the antibiotic concentration in POPC/ERG (67/33 mol.%) and POPC/CHOL (67/33 mol.%) liposomes in the absence and presence of phloretin, biochanin A, and genistein. (**b**) The ratio of amphotericin B absorbance at 363 and 427 nm characterizing the balance between self-associated and monomeric antibiotic forms in the distinct lipid microenvironment in the absence and presence of phytochemicals. Inset*:* The representative amphotericin B absorbance spectrums in the absence (control) and presence of phloretin in the POPC/ERG (67/33 mol.%) vesicles.

First, we checked if the effects are related to the alteration in the partitioning of the antibiotic between the aqueous and membrane phases. To compare the AmB concentration in the untreated POPC/ERG-liposomes and after the introduction of the most effective potentiator, phloretin, we performed absorbance spectroscopy measurements. The AmB absorbance spectrum in the lipid microenvironment revealed distinct maxima at 427, 399, 376, and 363 nm. According to [30,39], the first three maxima are typical for the solution of AmB in DMSO and are related to the monomeric form of the antibiotic, while the last one is due to the absorbance of AmB aggregates. Figure 4 demonstrates that the phloretin, biochanin A, and genistein did not practically affect the antibiotic absorbance spectrum, in particular, the optical density at 427 and 363 nm, indicating that these plant polyphenols did not alter the lipid/water partitioning of AmB and the balance between its monomeric and self-associated membrane forms. Thus, we concluded that the enhancement in AmB-produced transmembrane current by phloretin, biochanin A, and genistein was not related to the increase in the membrane concentration of polyene. The intersection of the absorption spectra of the antibiotic and the phytocompounds, which reduce the AmB pore-forming activity, cardamonin, 4′-hydroxychalcone, licochalcone A, butein, curcumin, and piperine [40,41,42,43,44,45], does not allow one to estimate the antibiotic concentration in the membrane in the presence of these compounds by the spectrophotometric method.

**Figure 5:**
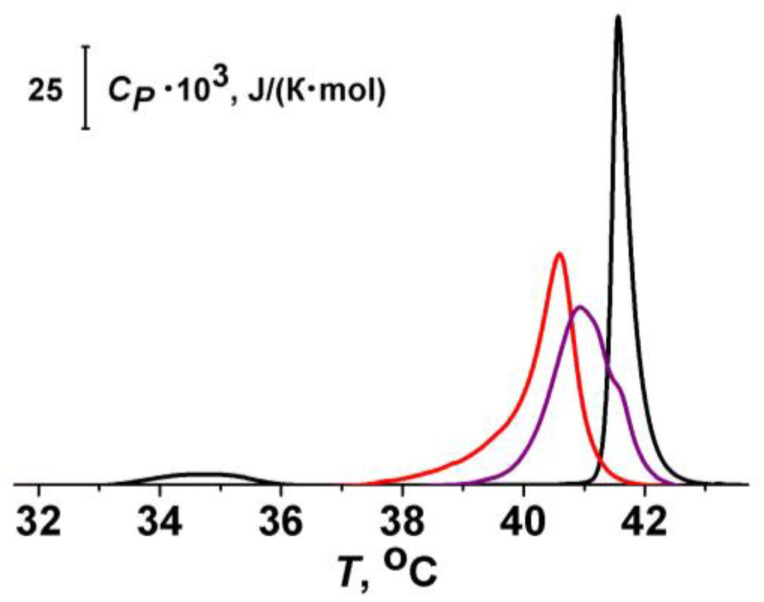
Heating thermograms of DPPC in the absence (black curve) and presence of phlorizin (purple curve) and biochanin A (red curve). The lipid:polyphenol molar ratio is equal to 10:1.

It is known that antifungal polyene macrolide antibiotics AmB and its close analog, nystatin, added to the lipid bilayer from one-side, form asymmetric single-length pores. The length of the pores is insufficient to penetrate the entire membrane [13,46]. Thus, polyene pore formation requires bending of the opposite lipid monolayer to form a lipid mouth of a pore having a positive curvature. This membrane deformation incurs an energetic cost that depends on bilayer material properties. Induction of local positive curvature stress in the membrane decreases the cost of pore formation and enhances the pore-forming activity of nystatin [31,38,47]. Based on the dependence of the polyene pores on curvature stress, we assessed whether the compounds could cause the changes in AmB-produced current by altering membrane elastics. Table 1 also summarizes the results of differential scanning microcalorimetry measurements of dipalmitoylphosphocholine melting after phytochemical addition. Figure 5 demonstrate the effects of phlorizin and biochanin A at the lipid:polyphenol molar ratio of 10:1 on the heating thermograms of DPPC. 

**Figure 6:**
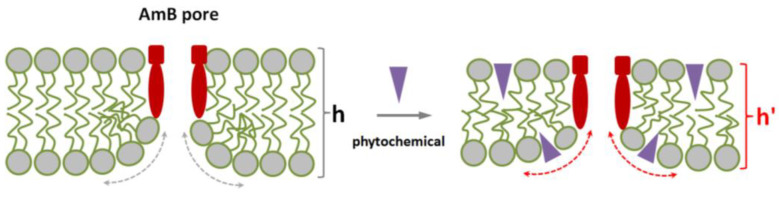
Scheme illustrating the facilitation of amphotericin B pore formation by the adsorption of plant small molecules on the lipid bilayer. Membrane lipids, pore-forming complexes (composed of amphotericin B and sterol), and phytochemical molecules are colored with grey, red, and violet respectively. h and h′ are the thicknesses of lipid bilayer in the absence and presence of phytochemical respectively, dotted curves indicates the positive curvature stress in the vicinity of lipid mouth of amphotericin B pore in the absence (grey) and presence of phytochemical (red) respectively.

One can notice that addition of phloretin, phlorizin, cardamonin, naringenin, taxifolin, biochanin A, resveratrol, 4′-hydroxychalcone, licochalcone A, butein, curcumin, quinine, colchicine, piperine, and dihydrocapsaicin led to a decrease in *T_m_* and an increase in ∆*T_b_* by more than 0.5 °C. Catechin, quercetin, and genistein slightly affected lipid melting temperature, but demonstrated a significant effect on phase transition peak width. Diminishment of the temperature and/or cooperativity of lipid melting indicated the disordering action of the tested phytochemicals on the membranes. More probably, these plant molecules are able to absorb on the membrane and intercalate between lipid heads, increasing the area per lipid molecule and causing membrane disordering. This suggests that the phytochemicals reduce the energetic cost of bilayer deformation. Thus, the action of phytochemicals on the lipid packing might explain the potentiating effects of phloretin, phlorizin, naringenin, taxifolin, biochanin A, resveratrol, quinine, and dihydrocapsaicin. The schematic representation of potentiating AmB pore formation by embedding of the phytochemicals between lipid heads into hydrophilic regions of the lipid bilayer is shown in Figure 6. A similar explanation was applied by Ingolfsson et al. to elucidate the alteration in gramicidin A pore-forming ability by capsaicin, curcumin, genistein, and resveratrol [48].

**Figure 7:**
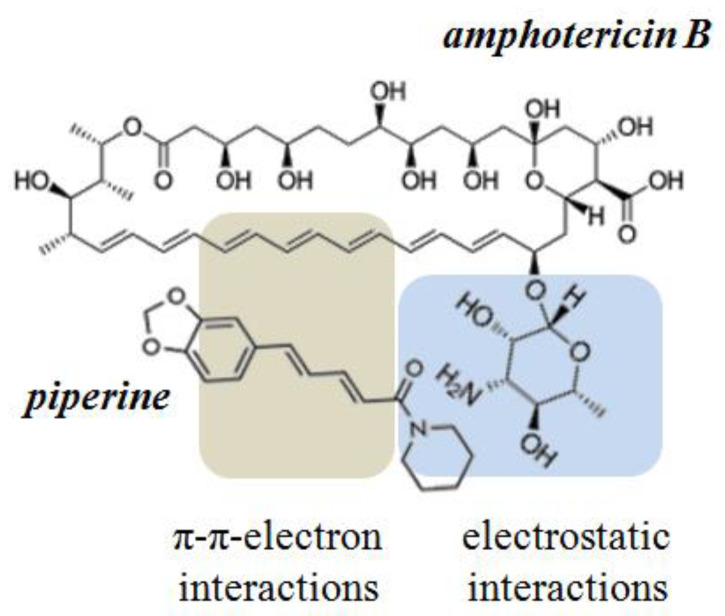
Schematic diagram of the intermolecular interactions between amphotericin B and piperine.

Considered in this way, the lack of effect of colchicine as well as the inhibitory effect of cardamomin, 4′-hydroxychalcone, licochalcone A, butein, curcumin, and piperine seems to be surprising. Comparing the structures of these phytochemicals one can notice that all of them share the same structural motif—a carbonyl group linked to a fragment composed of several conjugated double bonds. This motif is able to form both the electrostatic interactions with the mycosamine sugar residue and π–π electron interactions with the hydrophobic heptaene chain of AmB. It is possible that cardamomin, 4′-hydroxychalcone, licochalcone A, butein, curcumin, and piperine might compete with ERG for binding to the antibiotic and thus destroy the pore-forming AmB/ERG complexes. According to Neumann et al. [49], the complex between polyene and sterol molecules is stabilized by van der Waals interactions. The coplanarity and parallelism between polyene and sterol molecules determine the strength of their interaction. At the same time the hydrogen bonds formed between the sterol OH-group and AmB amino sugar residue control the relative orientation of the interacting molecules. The π–π electron interactions between double bonds in the steroid core and the side chain of the ERG molecule and polyene chain of AMB, additionally stabilize the AmB/ERG complex [50]. The possible influence of small molecules on the stability of polyene–sterol complexes was also discussed in [51,52]. The possibility of interaction between tested molecules having the indicated structural motif and AmB can modify the interaction of the polyene with the sterol and affect the stability of the pore-forming complexes. Figure 7 shows the schematic representation of intermolecular bonds between AmB and piperine molecules.

The sterol-dependent mode of action of polyene macrolides suggests that the specificity of antibiotics for different cells is determined by the sterol composition of their membranes. The preferred affinity of AmB for ERG controls the selectivity of its action on fungal cells. However, this selectivity is not absolute, AmB can interact with CHOL in membranes of mammalian cells; and this is the reason for the high toxicity of the antibiotic. In order to predict the possible toxicity, we tested the effects of phytochemicals on the pore-forming ability of AmB in lipid bilayers composed of POPC/CHOL (67/33 mol.%) (Table 1). Phlorizin, naringenin, quinine, and dihydrocapsaicin caused an increase in the pore-forming activity of AmB in the CHOL-containing bilayers by 3, 1.4, 1.6 and 1.7 times, respectively. Phloretin, catechin, taxifolin, quercetin, biochanin A, genistein, and resveratrol did not practically affect AmB-produced current in the CHOL-enriched membranes (*I_m_/I_o_* was about 1). Taking into account that the membrane lipid packing, and phase segregation depend on the type of membrane-forming sterol [53], a possible reason for the observed discrepancy between the effects of phytochemicals on the AmB pore-forming ability in ERG- and CHOL-enriched lipid bilayers might be related to a difference between elastic properties of these membranes. An increase in the pore-forming ability of AmB in ERG-containing membranes and the absence of the potentiating effect of phloretin, catechin, taxifolin, quercetin, biochanin A, genistein, and resveratrol on the polyene-induced current in CHOL-enriched bilayers (Table 1) can result in an increase in the antibiotic selectivity index. In silico evaluation of rat acute toxicity of identified potentiators of the pore-forming activity of AmB using GUSAR software [54] predicts low toxicity of the compounds with various routes of administration. Taking into account the in silico evaluation, the plant polyphenols are recommended for consideration in the development of new improved liposomal formulations of AmB. Thus, the approach used, based on the combination of AmB with plant molecules affecting membrane elasticity, can be applied not only to reduce the effective therapeutic concentration of the polyene, but also to expand its therapeutic window.

## 4. Conclusions

(1) Phloretin, phlorizin, naringenin, taxifolin, quercetin, biochanin A, genistein, resveratrol, and quinine are able to significantly potentiate the AmB pore-forming ability. This effect might be explained by membrane disordering. The replacement of ergosterol with cholesterol dramatically diminishes the potentiating effects of phloretin, taxifolin, quercetin, biochanin A, genistein, and resveratrol, demonstrating the possibility to enhance the selectivity index of the antibiotic.

(2) A combination of AmB with these polyphenols seems to be a promising approach to decrease antibiotic toxicity while maintaining its efficacy.

(3) Cardamomin, 4′-hydroxychalcone, licochalcone A, butein, curcumin and piperine are able to inhibit the AmB pore-forming ability, probably by binding the antibiotic.

For the first time, the question of why various compounds of different chemical structures and plant sources have a similar enhancing effect on the membrane activity of AmB has been investigated. Identified phytochemicals are able to alter membrane elasticity, which leads to a decrease in the energy cost of the formation of the lipid mouth of AmB pores. This opens a principal way of searching for new possible potentiators of membrane and, probably, antifungal antibiotic activity.

In addition, one way to regulate AmB activity has been discovered; binding of plant molecules containing a certain structural motif. It should be noted that the production of compounds with a similar structural motif, asperjinone and terrain, by *Aspergillus terreus* [55] might be an explanation for the refractory nature of these fungi to AmB therapy.

## Figures and Tables

**Table 1:** The effects of plant metabolites on the amphotericin B pore-forming activity and dipalmitoylphosphocholine main phase transition.

Phytochemical	*I_m_/I_o_*		−Δ*T_m_*, °C	ΔΔ*T_b_*, °C
	POPC/ERG	POPC/CHOL		
phloretin	18.1 ± 14.0	0.9 ± 0.1	1.2 ± 0.3 [34]	5.1 ± 0.7 [34]
phlorizin	4.3 ± 1.1	3.2 ± 0.6	0.6 ± 0.1	0.6 ± 0.2
cardamonin	0.7 ± 0.2	*n.d.*	1.5 ± 0.6 [35]	2.4 ± 0.5 [35]
naringenin	2.7 ± 0.5	1.4 ± 0.5	1.3 ± 0.2 [35]	1.9 ± 0.2 [35]
catechin	1.7 ± 0.2	1.0 ± 0.1	0.3 ± 0.1 [34]	1.3 ± 0.2 [34]
taxifolin	3.5 ± 1.3	1.1 ± 0.1	0.9 ± 0.1 [34]	1.4 ± 0.2 [34]
quercetin	6.2 ± 2.6	1.1 ± 0.1	0.3 ± 0.1 [36]	3.2 ± 0.3 [36]
biochanin A	11.9 ± 6.7	0.8 ± 0.1	0.9 ± 0.1	2.7 ± 0.3
genistein	9.8 ± 4.4	0.9 ± 0.1	0.2 ± 0.1 [34]	0.7 ± 0.1 [34]
resveratrol	6.7 ± 1.9	0.9 ± 0.2	1.9 ± 0.2 [35]	2.3 ± 0.4 [35]
4′-hydroxychalcone	0.7 ± 0.2	ND	2.0 ± 0.5 [35]	4.9 ± 0.6 [35]
licochalcone A	0.6 ± 0.1	ND	1.8 ± 0.1 [35]	4.5 ± 0.4 [35]
butein	0.4 ± 0.1	ND	2.3 ± 0.5 [35]	5.1 ± 0.4 [35]
curcumin	0.5 ± 0.1	ND	2.6 [37] *	8.6 [37] *
quinine	4.9 ± 2.9	1.6 ± 0.2	0.6 ± 0.1 [38]	0.8 ± 0.1 [38]
colchicine	1.1 ± 0.3	ND	0.8 ± 0.3 [38]	1.9 ± 0.3 [38]
piperine	0.4 ± 0.1	ND	2.9 ± 0.3 [38]	3.4 ± 0.2 [38]
dihydrocapsaicin	1.9 ± 0.3	1.7 ± 0.2	4.1 ± 0.2 [38]	4.5 ± 0.4 [38]

*I_m_/I_o_*—a ratio of the amphotericin B produced transmembrane current after and before the addition of the phytochemical at 50 mV. Membranes were composed of POPC/ERG or POPC/CHOL (67/33 mol.%) and bathed by 2.0 M KCl (pH 7.4). The concentration of phloretin, phlorizin, cardamonin, naringenin, catechin, taxifolin, quercetin, resveratrol, 4′-hydroxychalcone, licochalcone A, butein, and curcumin in the bilayer bathing solution was equal to 100 µM; the concentration of quinine, colchicine, piperine, and dihydrocapsaicin was equal to 400 µM. Δ*T_m_*, ΔΔ*T_b_*—phytochemical-induced changes in the main phase transition temperature of dipalmitoylphosphocholine and the width of the peak corresponding to lipid melting at the lipid:phytochemical molar ratio of 10:1; *—according to [37] at the lipid/curcumin molar ratio of 16:1. ND—not determined due to absence of pharmacological perspective for combination with antibiotics.
